# Efficient Removal of Volatile Organic Compounds by FAU-Type Zeolite Coatings

**DOI:** 10.3390/molecules25153336

**Published:** 2020-07-23

**Authors:** Mathieu Diboune, Habiba Nouali, Michel Soulard, Joël Patarin, Guillaume Rioland, Delphine Faye, T. Jean Daou

**Affiliations:** 1 Institut de Science des Matériaux de Mulhouse (IS2M), CNRS, Axe Matériaux à Porosité Contrôlée (MPC), Université de Haute-Alsace (UHA), 3 bis rue Alfred Werner, 68093 Mulhouse, France; mathieu.diboune3@uha.fr (M.D.); habiba.nouali@uha.fr (H.N.); 2 Université de Strasbourg, 67000 Strasbourg, France; 3 Service Laboratoires & Expertise, Centre National d’Etudes Spatiales (CNES), 18 avenue Edouard Belin, CEDEX 9, 61401 Toulouse, France; guillaume.rioland@cnes.fr (G.R.); delphine.faye@cnes.fr (D.F.); 4 Zéphir Alsace, 15 rue des Frères Lumière, 68350 Brunstatt-Didenheim, France; soulard.michel.za@orange.fr (M.S.); joel.patarinza@gmail.com (J.P.)

**Keywords:** molecular decontamination, FAU-type zeolite, silicone resins, zeolite coatings, organic pollutant adsorption

## Abstract

Silicone and pure organic binders were used to develop FAU-type zeolite coatings applied on pre-treated aluminum substrates by using a spraying method and then cured under specific conditions. The influence of the amount of binder on adhesion properties of zeolite coatings was first investigated to determine the optimum ratio between zeolite and binder. Zeolite coatings were then elaborated with a high zeolite content (between 70 and 80 wt.%) to ensure high adsorption capacities. The amount of binders involved in different zeolite coatings was sufficient to achieve interesting adhesion and cohesion properties. The accessibility of zeolite microporosity was studied by nitrogen adsorption-desorption measurements, which revealed a very small or no loss of the micropore volume for the optimized coatings. Volatile Organic Compounds (VOCs) adsorption measurements were carried out using *n*-hexane as probe molecule. FAU-type zeolite in powder form adsorbs 180 mg/g_anhydrous zeolite_, whereas the amounts of *n*-hexane adsorbed by zeolite coatings ranged from 131 to 175 mg/g_anhydrous zeolite_.

## 1. Introduction

Silicone resins are used in various applications since they have been discovered by Eugene G. Rochow in 1941 [1]. They consist of a framework Si-O-Si where organic groups are bonded to the silicon atom [2]. Due to their high thermal stability in comparison with common organic polymers made with carbon-carbon bonds [3], silicone resins are of particular interest for high-temperature applications [4,5,6,7]. Generally, they are stable at temperatures up to 250 °C over a long period. This high thermal stability is due to the physicochemical properties of the siloxane bond (Si-O-Si), which shows a high bonding energy. The one of Si-O bond (460.5 kJ/mol) is higher than those of C-O (358 kJ/mol) and C-C (304 kJ/mol) bonds [8]. Moreover, in addition to high thermal stability, silicone resins combine also the flexibility of common organic polymers, which allows them to adhere to a large number of surfaces [9]. These properties make silicone resins very interesting candidates for the space industry where the temperature can vary between −110 and 150 °C under orbital conditions [10]. In addition to the drastic conditions encountered in orbit, satellites are subjected to shocks and vibrations, especially at launch. Thus, selected compounds must be stable in this temperature range and under mechanical stress. In space, any trace of contamination due to compounds degradation must be avoided. Indeed, global performances of satellites in low Earth orbit are already affected by the phenomenon of on-orbit molecular contamination generated by the outgassing of organic pollutants emanated from spacecraft polymers under orbital conditions. The outgassing of these molecules leads to their deposition on sensitive surfaces such as thermal control surfaces, detectors, electronic and optical devices, which results in a drastic decrease of their thermal and/or optical properties. Molecules such as plasticizers and hydrocarbons were found to be mainly responsible of this molecular contamination by the National Aeronautics and Space Administration (NASA) and the French Space Agency (CNES) [11,12]. According to the European Cooperation for Space Standardization (ECSS) created in 1993, various techniques are recommended to reduce the outgassing of organic pollutants in satellites on orbit such as strict criteria for materials selection or a preliminary bakeout of spacecraft materials [13]. However, the use of low outgassing materials remains very costly and spacecraft materials continue to outgas in orbit even if a preliminary bakeout was performed. Consequently, the use of molecular adsorbents has been considered. The project Wide Field/Planetary Camera-2 (WFPC-2) conducted by NASA in 1994 allowed to select zeolites as the most efficient adsorbent materials for trapping and retention of organic pollutants in orbit [14] because of their ability to trap organic molecules at very low pressure and very low concentration [15]. Moreover, their high thermal and mechanical stability are also of particular interest for space application. 

Among the 252 different structural types of zeolites referenced by the Structural Commission of the International Zeolite Association (IZA), the FAU-type structure is one of the most widely used for industrial applications. FAU-type zeolites are one of the main components of cracking catalysts due to their structure [16]. The structure is composed of supercages interconnected through a circular 12-member-ring (MR) with a pore opening of 7.4 Å. The FAU-type structure is used for several applications such as the separation of hydrocarbon and gas mixtures [17,18], catalysis [19,20] and D_2_/H_2_ adsorption selectivity [21]. FAU-type structure was used in this work mainly due to the large pore opening and also because of its high adsorption capacities of Volatile Organic Compounds (VOCs) [15,22,23].

Unfortunately, conventional syntheses of FAU-type zeolite give rise generally to a fine crystalline powder that is problematic to use as it is for molecular decontamination applications, especially in satellites. In fact, a secondary dust contamination could occur due to zeolite particles spreading caused by shocks and vibrations during satellite takeoff. The shaping of zeolites is then required to avoid any risk of particulate contamination. Therefore, zeolites were successfully shaped by CNES into films, pellets and beads [10,22,23,24,25,26] with the best mechanical and adsorption properties obtained in the case of films and pellets. However, the major drawbacks of these shaping methods are the difficulties to incorporate them into satellite/spacecraft design (special design needed for shaped zeolites integration) and the amount of zeolites that can be incorporated. That is why zeolite coatings have been developed in this study because they can be applied directly to satellite surfaces by a spraying method without additional modifications on satellites.

Zeolite powders are usually shaped with the addition of one or several binders to improve mechanical performances of the final zeolitic object. A wide range of organic and inorganic binders have been used in combination with zeolites to shape them and enhance their mechanical properties [27,28,29,30,31,32]. Regarding zeolite coatings, inorganic binders (colloidal suspensions LUDOX^®^) were employed by NASA to develop zeolite coatings for organic pollutants trapping in orbit [33,34]. Silicone resins were also used in the elaboration of zeolite coatings for thermal applications and good bonding performances were obtained on different aluminum alloy substrates [9,35,36]. To avoid a drastic decrease of adsorption capacities, the ratio between quantities of zeolite and binder is important to control. Indeed, the addition of a binder may lead to an obstruction of the zeolite porosity and therefore to a decrease of zeolitic materials adsorption capacities [37,38].

In this study, different commercial binders (five silicone binders and one pure organic binder) were used in combination with 13X zeolite powder and the appropriate solvent (water or *m*-xylene) to develop zeolite coatings with high zeolite content and good adsorption and mechanical properties. Binders were selected depending on several parameters: curing conditions (high and room temperature, with or without the use of a catalyst) and solubility in solvents (water or *m*-xylene). The use of water as solvent is a real advantage since it does not contribute to the emission of VOCs in the atmosphere. Zeolite coatings were applied on aluminum alloy substrates (used in aerospace) by using a spraying method and then cured under the ideal conditions specific to each binder. 

Adhesion, textural and adsorption properties regarding VOCs of the elaborated zeolite coatings will be discussed. *n*-Hexane is used as probe molecule to simulate the organic volatile pollutant because it is one of the contaminants identified by NASA and CNES. This probe molecule is easy to handle and has often been used to study adsorption capacities of organic pollutants by zeolites [39,40].

## 2. Results

### 2.1. Characterization of the Reagents

**Figure 1:**
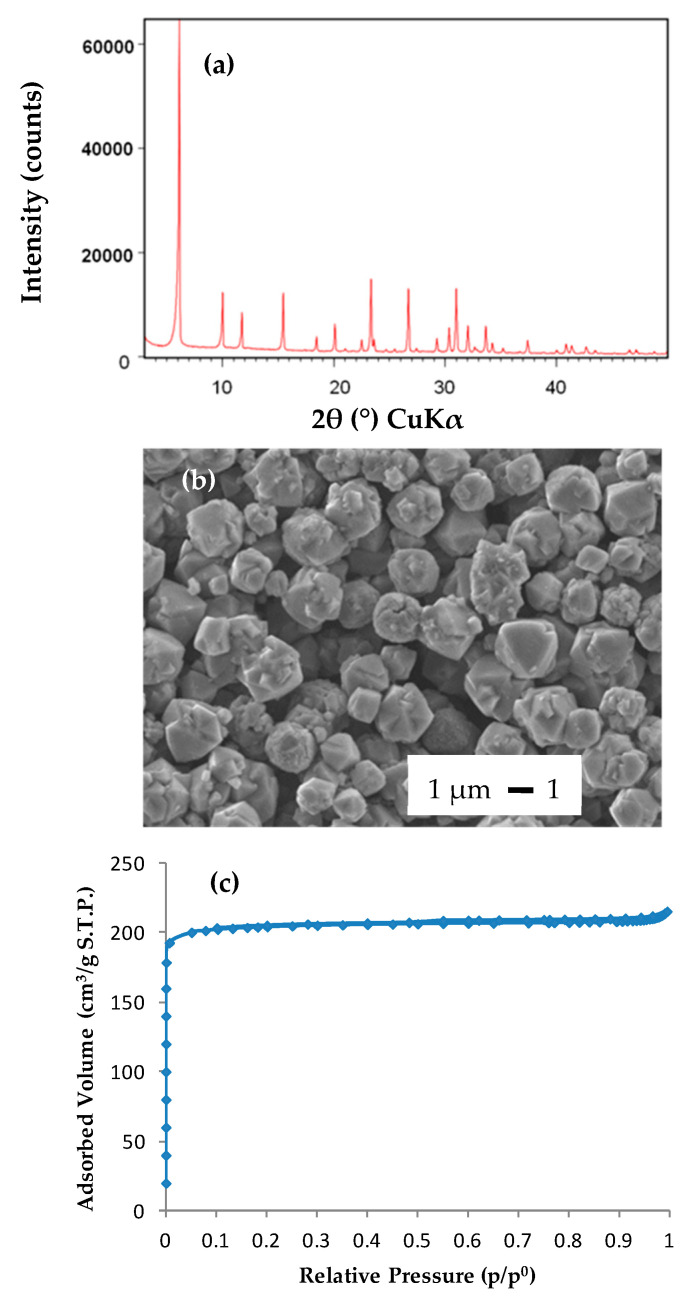
Pure 13X zeolite powder (FAU): (**a**) X-ray diffraction (XRD) pattern; (**b**) scanning electron micrograph; (**c**) nitrogen adsorption-desorption isotherms at −196 °C.

13X zeolite was characterized by X-ray diffraction (XRD), scanning electron microscopy and nitrogen adsorption-desorption. The XRD pattern (Figure 1a) presents characteristic peaks of pure FAU-type zeolite. The scanning electron micrograph shows a bipyramidal morphology of the crystals, which is characteristic of FAU-type phase (Figure 1b). The particles size is of 2–3 µm. The good crystallization rate of FAU 13X zeolite is also confirmed by nitrogen adsorption-desorption isotherms of type I characteristics of microporous materials according to the International Union of Pure and Applied Chemistry (IUPAC) classification [41]. Indeed, the experimental micropore volume (0.30 cm^3^·g^−1^) is in good agreement with values of pure FAU-type zeolite reported in literature (Figure 1c) [22,23,42].

**Figure 2:**
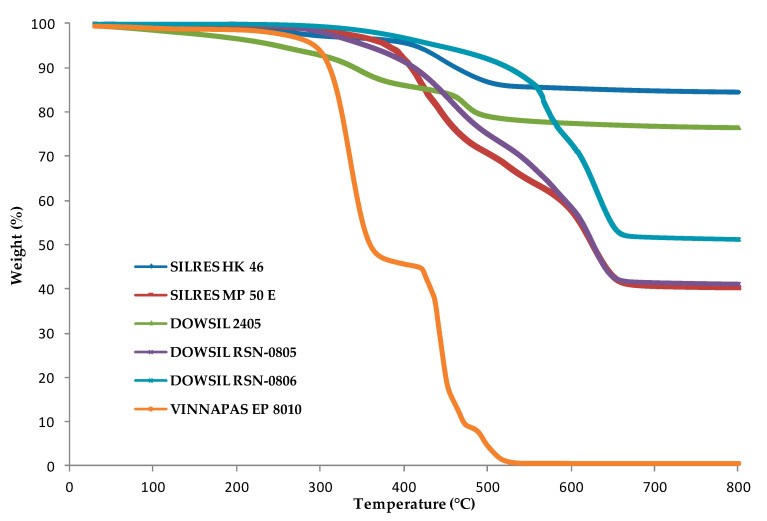
TGA (Thermogravimetric analyses) curves of commercial polymerized binders showing the weight loss as a function of the temperature (°C).

In space environment, the temperature typically varies from −110 to 150 °C. Commercial binders must not decompose in this range of temperature. That is why commercial binders were characterized by thermogravimetric analysis (Figure 2) in order to determine their maximum temperature of use. All binders were first polymerized before being analyzed. Silicone resins used in this work are composed of alkoxy silanes with highly reactive silanol groups, which condense themselves to form oligomeric siloxane structures above 200 °C (except for DOWSIL^®^ 2405) or at room temperature with the use of a catalyst (DOWSIL^®^ 2405), whereas VINNAPAS^®^ EP 8010 is a pure organic resin. This resin is an aqueous polymer dispersion based on the monomers vinyl acetate and ethylene which polymerize at room temperature. Temperatures given in Table 1 correspond to the first weight loss of the polymerized binders and do not always correspond to the temperature from which decomposition begins. The weight loss may correspond to the evaporation of residual volatile compounds present in the binders or to the polymerization process. In all cases, no weight loss is observed at a temperature below 250 °C except for the binder DOWSIL^®^ 2405 (see Table 1). Nevertheless, only a small weight loss is observed between 100 and 200 °C for this binder and it does not correspond to its decomposition due to the absence of any sign of degradation for FAU-2405 coating. Except for the organic binder VINNAPAS^®^ EP 8010, which begins to degrade itself at 300 °C, the remaining mass stable at a temperature of 800 °C of each silicone binder corresponds to amorphous silica. It is possible to conclude that all binders (the monomer and the polymerized version) selected in this study are stable at a temperature of at least 150 °C. Therefore, they will not be affected by the curing conditions and the activation procedure (150 °C under high vacuum for 15 h) used before nitrogen adsorption-desorption experiments.

### 2.2. Influence of the Amount of Binder on Adhesion Properties

**Figure 3:**
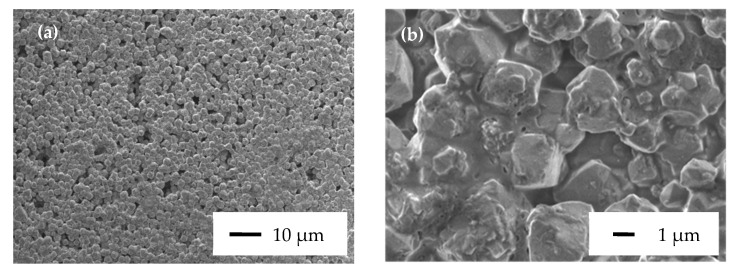
SEM micrographs of the zeolite FAU-HK46 coating 4 made with a zeolite content of 80 wt.% at different scales (**a**) 10 µm; (**b**) 1 µm.

The influence of the amount of the binder on adhesion properties of zeolite coatings was studied. The purpose of this experiment was to find out the maximum amount of zeolite that is possible to incorporate in a coating in order to have the highest possible adsorption capacities without affecting the mechanical and especially the adhesion properties of the coating for the targeted application. Experiments were performed on zeolite coatings made with the commercial binder SILRES^®^ HK 46 with five different binder/zeolite ratios (in wt.%). All samples were characterized by the cross-cut test and results as well as coatings composition after curing are listed in Table 2. The amount of zeolite varies from 50 to 90 wt.% regarding the total weight of the final dry coating after the evaporation of the solvent and the binder crosslinking reaction. The results presented in Table 2 show interesting adhesion properties for zeolite coatings 1 to 4 with an adhesion note of 0 according to ISO 2409 classification. For zeolite coating 5, an adhesion note of 3 was obtained due to significant cracks observed after the cross-cut test (Figure S1). That is why zeolite coating 4 is the right compromise between low but sufficient amount of binder to maintain good adhesion and cohesion properties and high amount of zeolite to achieve good adsorption properties. The presence of the binder is visible between zeolite crystals on SEM micrographs (Figure 3) and the quantity of binder involved is sufficient to bind zeolite particles together and to ensure a good adhesion between the coating and the aluminum substrate. This coating was developed with a binder/zeolite ratio of 40 wt.% before the curing procedure and this ratio was fixed for all other coatings which will be described later in this study. This ratio of 40 wt.% corresponds to a binder/zeolite ratio of 25 wt.% after the curing procedure for FAU-HK46 coating where binder and zeolite contents are of 20 and 80 wt.% in the final dry coating, respectively. Zeolite and binder contents were calculated by taking into account the solid content of the resin (after the crosslinking process) and the mass of the dehydrated 13X zeolite. Due to the solvent evaporation, the coating FAU-HK46 contains only these two compounds after curing. For example, the silicone resin SILRES^®^ HK 46 has a solid content of 50 wt.% and an amount of 23 wt.% of water is adsorbed by 13X zeolite powder. For all samples, which will be described later, each solid content is calculated after curing according to the following method:

$$\mathrm{Zeolite}\;\mathrm{content}=\frac{m_{dehydrated\;zeolite}}{m_{total}\;}\;\;\;\mathrm{Binder}\;\mathrm{content}=\frac{m_{post-crosslinking\;binder}}{m_{total}}$$

where m_total_ is corresponding to the total mass of the final coating after the curing procedure. The total mass includes the dehydrated zeolite, the binder after crosslinking and the catalyst if used.

### 2.3. Influence of the Nature of the Binder on the Mechanical and Adsorption Properties of the Final Zeolite Coating

**Figure 4:**
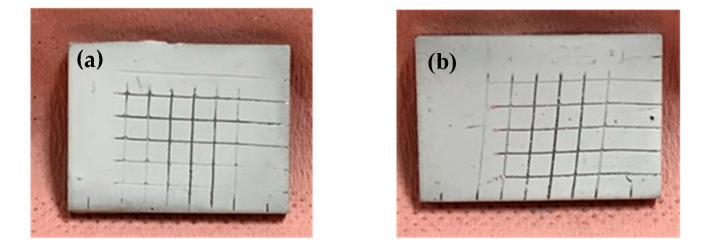
Adhesion test performed on zeolite coatings: (**a**) FAU-HK46; (**b**) FAU-RSN0805.

The binder is the essential constituent of the coating to provide good adhesion between the coating and the substrate and also cohesion properties between zeolite crystals to avoid any cracks in the coating layer. The characteristics of each zeolite coating as well as results of cross-cut tests and curing conditions are summarized in Table 3. Despite the large amount of zeolite involved in each zeolite coating, amounts of binder between 20 and 25 wt.% were sufficient to obtain adhesion notes of 0 and 1 showing good adhesion properties of these coatings on aluminum substrates. Indeed, less than 5% of the total surface area of the coating is detached after the cross-cut test as it is possible to see on zeolite coatings FAU-HK46 and FAU-RSN0805 in Figure 4. The difference between adhesion notes of 0 and 1 is really not significant. Coating thicknesses of less than 60 µm were measured but it is difficult to control the film thickness of each coating due to the method of deposition used in this work. For example, a lack of homogeneity is observed for FAU-MP50E coating where the thickness varies from 10 to 40 µm on the same substrate. No sign of flaking is observed on zeolite coatings except for FAU-EP8010 coating, where the formation of a thin and transparent film is observed on the surface which can be removed very easily. Nevertheless, results show overall good bonding performance on aluminum substrates enabling to obtain compatible adhesion properties for the targeted application.

In addition to promising adhesion properties, adsorption capacities of zeolite coatings must not be impacted by the presence of binders, which can lead to a partial clogging of the porosity. That is why it is essential to characterize adsorption capacities of zeolite coatings developed in this study.

**Figure 5:**
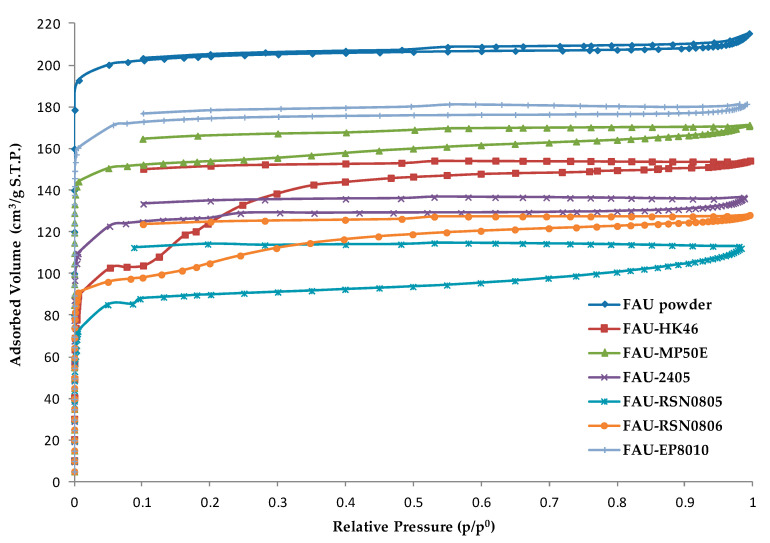
Nitrogen adsorption-desorption isotherms of pure 13X powder and zeolite coatings.

In order to investigate adsorption capacities of zeolite coatings, nitrogen adsorption-desorption experiments were carried out at −196 °C on the powder form of each coating (Figure 5). Pure 13X zeolite in powder form was also characterized by nitrogen adsorption-desorption to use it as a reference to determine the microporosity accessibility of zeolite coatings. Micropore volumes and values of porosity accessibility for zeolite coatings are summarized in Table 4 and they were determined on desorption isotherms. Nitrogen adsorption-desorption experiments were performed on powder mixture form of all samples. These powders were obtained using a part of coating slurries that were used for the elaboration of the corresponding zeolite coatings. These samples were then cured in the same conditions applied for zeolite coatings. After the curing procedure, a grinding operation was performed with a mortar and a pestle to obtain a quite fine powder to be analyzed. For FAU-HK46, FAU-MP50E, FAU-2405 and FAU-EP8010 coatings, the amount of binder (between 20 and 25 wt.%) has no influence on adsorption capacities (see Table 4). Indeed, micropore volumes for all coatings (for 100% of zeolite) range from 0.29 to 0.30 cm^3^·g^−1^. These values are similar to the one obtained for pure FAU-type powder (0.30 cm^3^·g^−1^), meaning that binders SILRES^®^ HK 46, SILRES^®^ MP 50 E, DOWSIL^®^ 2405 and VINNAPAS^®^ EP 8010 do not block the access to micropores. Concerning FAU-HK46 coating, the adsorption phase takes place in two phases. This phenomenon is observed in the range of low relative pressures (0.1 < p/p^0^ < 0.3) and it is repeatable and specific to this zeolite coating (Figure 5). For polymerized SILRES^®^ HK 46 binder, no adsorption of N_2_ is observed (Figure S2), a plausible explanation for this behavior would be a possible interaction between this binder and 13X zeolite which slow down the N_2_ uptake in the microporosity. For FAU-RSN0806 and FAU-RSN0805 coatings, the adsorption capacities are reduced by 20 and 30% compared to pure 13X zeolite powder, respectively. Nevertheless, despite the partial clogging to the porosity access due to the use of both binders DOWSIL^®^ RSN-0806 and DOWSIL^®^ RSN-0805, the porosity remains mostly accessible. 

Globally, amounts of binders ranging from 20 to 25 wt.% depending on the zeolite coating were sufficient to enable good adhesion, cohesion and adsorption properties. In order to simulate adsorption capacities of a specific organic pollutant by FAU-type zeolite coatings, *n*-hexane adsorption experiments were performed for each coating.

The *n*-hexane molecule was used as probe molecule to simulate adsorption capacities of zeolite coatings with respect to a specific organic pollutant. *n*-Hexane adsorption experiments were performed inside a desiccator preliminary dried to remove moisture. To determine the adsorbed amount of *n*-hexane, weighings of each sample were carried out before the start of the experiment and afterwards the samples have been placed for 24 h in a desiccator under an atmosphere of *n*-hexane at room temperature. Only two weighings were performed to minimize water co-adsorption by zeolite coatings due to the use of the hydrophilic FAU-type zeolite. An experiment time of 24 h was enough to reach the saturation stage. All *n*-hexane adsorption capacities of each coating in powder form are reported in Table 5. All samples were characterized three times. Adsorbed amount of *n*-hexane in FAU-type powder obtained in this work (180 mg/g_anhydrous zeolite_) is in good agreement with values reported in literature ranging from 181 to 186 mg/g_anhydrous zeolite_ [15,39]. Adsorbed amounts of *n*-hexane between 131 and 175 mg/g_anhydrous zeolite_ were measured for all zeolite coatings in powder form (Table 5). Adsorbed amounts of *n*-hexane are globally coherent with the accessible microporous volume determined by nitrogen sorption measurements (Table 4) except for FAU-MP50E, FAU-2405 and FAU-EP8010 coatings for which values are 10 to 15% lower than expected. Nevertheless, all zeolite coatings exhibit interesting adsorption capacities regarding the *n*-hexane molecule, which are promising for the intended application. *n*-Hexane adsorption experiments (done in a desiccator) were also performed on zeolite coatings applied on aluminum substrates and the same results were obtained, thus confirming that the binder does not affect the adsorption capacities of the zeolite. FAU-type zeolite coatings remain very interesting due to their high adsorption capacities and also because the FAU-type structure has a pore opening of 7.4 Å, which gives it the ability to trap organic pollutants with a larger kinetic diameter than the one of *n*-hexane, which is 4.3 Å [15,22,23].

## 3. Conclusions

FAU-type zeolite coatings were elaborated by mixing, separately, different commercial binders (mostly silicone resins) with the appropriate solvent, 13X powder and a titanate catalyst, if necessary, to catalyze the crosslinking reaction. Cross-cut tests showed that adhesion properties are very interesting despite the small amount of binder used in zeolite coatings (between 20 and 25 wt.%).

Nitrogen adsorption-desorption experiments confirmed that the microporosity remains totally or almost totally accessible for the zeolite coatings developed in this study.

Adsorption of *n*-hexane experiments showed that zeolite coatings exhibit interesting adsorption properties with adsorbed amounts ranging from 131 to 175 mg/g_anhydrous zeolite_. These results show that these zeolite coatings possess high potential for on-orbit molecular decontamination.

## 4. Materials and Methods 

### 4.1. Materials

13X powder with a crystal size of 2–3 μm and a Si/Al molar ratio of 1.2 was purchased from Sigma-Aldrich (Saint Louis, MO, USA). Commercial binders were supplied from IMCD and Univar (Downers Grove, IL, USA) and the catalyst tetrabutyl titanate was purchased from Sigma-Aldrich and were used as received. *n*-Hexane (with less than 0.01 wt.% of water, 95%) was purchased from Carlo Erba (Chaussée du Vexin, Val-de-Reuil, France) and was used as received as probe molecule. Sheets of aluminum alloy Al 6061 (300 × 300 × 2.3 mm^3^) were supplied by Alfa Aesar (Ward Hill, MA, USA) and they were cut into small substrates of 20 × 15 × 2.3 mm^3^ using a mechanical press.

### 4.2. Characterization Techniques

The X-Ray diffraction pattern was recorded on a PANalytical MPD X’Pert Pro diffractometer (Almelo, The Netherlands) operating with Cu Kα radiation (Kα = 0.15418 nm) equipped with a PIXcel 1D detector (active length = 3.347°2θ). The XRD powder pattern was collected at ambient temperature in the 3° < 2θ < 50° range, by steps of 0.013° in 2θ and with a time of 220 s by step. The Si/Al molar ratio of FAU-type zeolite was determined using an X-Ray fluorescence spectrometer PANalytical Zetium (4 kW). A scanning electron microscope JEOL JSM-7900F (Tokyo, Japan) was used to study morphological properties of the pure zeolite FAU 13X and zeolite coatings. Thermogravimetric analyses were carried out under air with a Mettler-Toledo TGA/DSC 1 (Greifensee, Switzerland) between room temperature and 800 °C at a heating rate of 5 °C·min^−1^. Coatings thickness measurements were carried out by using an ISOSCOPE^®^ FMP10 equipped with a probe FTA3.3H purchased from Fischer (Sindelfingen, Germany) allowing the measurement of coating thickness on non-ferrous substrates. Adhesion properties of zeolite coatings applied on aluminum support were characterized by performing cross-cut tests. The equipment used was a cutting knife fitted with parallel blades purchased from TQC (Capelle aan den Ijssel, The Netherlands). A right-angle lattice pattern was cut into the coating penetrating through to the substrate. A tape was then placed over the grid and removed to evaluate qualitatively the detachment of the coating from the substrate. The resistance of the coating to separation of the substrate was evaluated using the ISO 2409 classification ranging from 0 for an intact coating to 5 for a coating peeling off completely from the substrate. Textural properties of the 13X zeolite and zeolite coatings were determined from nitrogen adsorption-desorption isotherms performed at −196 °C with a Micromeritics ASAP 2420 (Norcross, GA, USA) instrument. Before each measurement, the samples were outgassed under vacuum at 150 °C for 15 h to ensure that the volatile materials adsorbed inside the porosity of the adsorbent material are driven off. The equilibrium time has been fixed at 30 s for each adsorption–desorption experiment. These experiments were used to determine the accessibility of zeolite coatings porosity compared to pure zeolite in powder form. The micropore volume of pure FAU powder was determined by using the *t*-plot method. Adsorption capacities of organic pollutants by zeolitic materials were studied at room temperature under *n*-hexane atmosphere inside a desiccator preliminary dried under argon flow to remove moisture. The molecule of *n*-hexane is one of the contaminants identified by NASA and CNES in satellites. Samples were previously activated under high vacuum at 150 °C for 15 h to remove all traces of volatile compounds from the porosity. 

### 4.3. Preparation of Zeolite Coatings

Sheets of aluminum alloy Al 6061 (20 × 15 × 2.3 mm^3^) were first treated by physical abrasion using emery paper (with 180 μm grit) for approximately 60 s and then washed with ethanol and acetone to remove any contaminants from the surface.

The 13X zeolite was saturated with water using a desiccator containing a saturated solution of ammonium chloride NH_4_Cl to keep the atmosphere at a constant relative humidity of 80% to avoid the variation of the mass of the zeolite during the different weighings. The amount of water adsorbed was determined by thermogravimetric analysis and found to be 23 wt.%.

All commercial binders, their characteristics and appropriate solvents are listed in Table 1. Each coating slurry was prepared by dispersing the hydrated zeolite in the appropriate solvent (water or *m*-xylene) by using an ultrasonic bath at room temperature for 10 min in order to disperse zeolite particles in the solvent to obtain a homogeneous suspension. The solvent content was fixed at 50 wt.% regarding the total weight of the suspension. Then, the binder (as purchased in solution, see Table 6) was added to the mixture of hydrated zeolite and solvent respecting a binder/zeolite ratio of 40 wt.% before the curing procedure. This ratio was fixed after a study on the influence of the quantity of binder on the adhesion properties of the zeolite coating applied on aluminum substrates using SILRES^®^ HK 46 as binder as will be shown below. It is important to notice that the amount of zeolite varies from 70 to 80 wt.% with regards to the total weight of the coating after the drying step, i.e., after the solvent has been evaporated. Depending on the binder used, a catalyst could also be used to catalyze the crosslinking reaction forming the polymer network at room temperature. After adding the binder and, if necessary, the catalyst, the slurry was then stirred one more time with an ultrasonic for 10 min to obtain a homogeneous formulation liquid enough to be spread on the aluminum substrate with the airbush pistol AFC-101A from Conrad Electonics. The wet coating was then cured according to the drying conditions detailed in Table 6.

## Figures and Tables

**Table 1:** Temperature of the first weight loss observed for the polymerized commercial binders by TGA.

Binder	Temperature of the First Weight Loss (°C)
SILRES^®^ HK 46	250
SILRES^®^ MP 50 E	300
DOWSIL^®^ 2405	100
DOWSIL^®^ RSN-0805	300
DOWSIL^®^ RSN-0806	350
VINNAPAS^®^ EP 8010	250

**Table 2:** Composition and adhesion note of zeolite coatings made with the 13X zeolite and the commercial binder SILRES^®^ HK 46 after thermal curing at 250 °C for 30 min.

Zeolite Coating	13X Zeolite Content(wt.%) ^1^	Binder SILRES^®^ HK 46 Content(wt.%) ^1^	Adhesion Note ^2^
1	50	50	0
2	60	40	0
3	70	30	0
4	80	20	0
5	90	10	3

^1^ Both zeolite and binder contents were calculated for the final dry coating. ^2^ Adhesion notes according to ISO 2409 classification are ranging from 0 for an intact coating to 5 for a coating peeling off completely from the substrate.

**Table 3:** Composition, adhesion note, coating thickness and curing conditions of zeolite coatings.

Zeolite Coating Code	13X Zeolite Content(wt.%) ^1^	Binder SILRES^®^ HK 46 Content (wt.%) ^1^	Adhesion Note ^2^	Coating Thickness (µm) ^3^	Curing Conditions
FAU-HK46	80	20	0	46 ± 2	250 °C/30 min
FAU-MP50E	80	20	0	28 ± 11	250 °C/30 min
FAU-2405	70	25	1	46 ± 7	RT/1 h (with catalyst) ^4^
FAU-RSN0805	80	20	0	33 ± 5	250 °C/30 min
FAU-RSN0806	80	20	0	46 ± 7	250 °C/30 min
FAU-EP8010	75	25	1	42 ± 6	RT/1 h

^1^ Both zeolite and binder contents were calculated for the final dry coating. ^2^ Adhesion notes according the ISO 2409 classification are ranging from 0 for an intact coating to 5 for a coating peeling off completely from the substrate. ^3^ Coating thicknesses were measured by using the ISOSCOPE^®^ FMP10 equipped with the probe FTA3.3H. An average value over 10 measurements on the same coating with the standard deviation is given. ^4^ 5 wt.% of the catalyst tetrabutyl titanate is present in the final dry coating FAU-2405.

**Table 4:** Micropore volumes and porosity accessibility of pure 13X zeolite and zeolite coatings determined from nitrogen adsorption-desorption isotherms at −196 °C.

Sample ^1^	13X Zeolite Content (wt.%) ^2^	Micropore Volume of Zeolite Coating in Powder Form (cm^3^·g^−1^)	Accessible Micropore Volume of Zeolite (cm^3^·g^−1^) ^3^	Porosity Accessibility (%) ^3^
FAU powder	100	0.30	0.30	100
FAU-HK46	80	0.23	0.29	95
FAU-MP50E	80	0.24	0.30	100
FAU-2405	70	0.21	0.30	100
FAU-RSN0805	80	0.17	0.21	70
FAU-RSN0806	80	0.19	0.24	80
FAU-EP8010	75	0.23	0.30	100

^1^ Nitrogen adsorption-desorption experiments were performed on the coating in powder form. ^2^ Zeolite content of each coating was calculated for the final dry coating. ^3^ Taking into account the amount of binders which does not adsorb N_2_.

**Table 5:** Adsorption capacities of *n*-hexane by pure 13X zeolite and zeolite coatings.

Sample ^1^	Adsorbed Amount of *n*-Hexane at Saturation Stage (mg/g_anhydrous zeolite_)
FAU powder	180 ± 2
FAU-HK46	175 ± 2
FAU-MP50E	164 ± 3
FAU-2405	160 ± 2
FAU-RSN0805	131 ± 3
FAU-RSN0806	141 ± 4
FAU-EP8010	152 ± 5

^1^*n*-hexane adsorption experiments were performed on the coating mixture in powder form.

**Table 6:** List of commercial binders, solid content, solvent, curing conditions and zeolite coating code.

Commercial Binder ^1^	Supplier	Solid Content (wt.%) ^2^	Solvent ^3^	Curing Conditions	Zeolite Coating Code ^4^
SILRES^®^ HK 46	IMCD	50	*m*-xylene	250 °C/30 min	FAU-HK46
SILRES^®^ MP 50 E	IMCD	50	water	250 °C/30 min	FAU-MP50E
DOWSIL^®^ 2405	Univar	72	*m*-xylene	RT/1 h (with catalyst) ^5^	FAU-2405
DOWSIL^®^ RSN-0805	Univar	50	*m*-xylene	250 °C/30 min	FAU-RSN0805
DOWSIL^®^ RSN-0806	Univar	50	*m*-xylene	250 °C/30 min	FAU-RSN0806
VINNAPAS^®^ EP 8010	IMCD	60	water	RT/1 h	FAU-EP8010

^1^ All binders are commercial silicone resins except the binder VINNAPAS^®^ EP 8010, which is an aqueous organic polymer dispersion. ^2^ Proportion of non-volatile material contained in the binder formulation after curing (dehydrated zeolite and binder without solvent). ^3^ Appropriate solvent recommended by the supplier for the formulation of coatings. ^4^ The code FAU refers to the structural type of the zeolite used in this study. ^5^ The addition of a catalyst (tetrabutyl titanate) is required by the supplier to cure the binder DOWSIL^®^ 2405 at room temperature (RT).

## Supplementary Materials

The following are available online at https://www.mdpi.com/1420-3049/25/15/3336/s1, Figure S1: Adhesion test performed on zeolite coatings FAU-HK46 1 to 5 (from left to right); Figure S2: Nitrogen adsorption-desorption isotherms of the polymerized commercial binder SILRES^®^ HK 46.
