# Supplementary material for: Efficient Removal of Volatile Organic Compounds by FAU-Type Zeolite Coatings
Source: Molecules. 2020 Jul 23;25(15):3336. doi: 10.3390/molecules25153336 (PMC7435809; doi:10.3390/molecules25153336)
Supplement: Supplementary file 1 [file molecules-25-03336-s001.pdf]

Supporting information

Article

Efficient removal of volatile organic compounds by FAU-type zeolite coatings

Mathieu Diboune [1,2,3], Habiba Nouali [1,2], Michel Soulard [4], Joël Patarin [4], Guillaume Rioland [3], Delphine Faye [3] and T. Jean Daou [1,2,*]

[1] Université de Haute-Alsace (UHA), CNRS, Axe Matériaux à Porosité Contrôlée (MPC), Institut de Science des Matériaux de Mulhouse (IS2M), 3 bis rue Alfred Werner, 68093 Mulhouse, France; mathieu.diboune3@uha.fr (M.D.); habiba.nouali@uha.fr (H.N.)
[2] Université de Strasbourg, 67000 Strasbourg, France
[3] Service Laboratoires & Expertise, Centre National d'Etudes Spatiales (CNES), 18 avenue Edouard Belin, 61401 Toulouse Cedex 9, France; guillaume.rioland@cnes.fr (G.R.); delphine.faye@cnes.fr (D.F.)
[4] Zéphir Alsace, 15 rue des Frères Lumière, 68350 Brunstatt-Didenheim, France; soulard.michel.za@orange.fr (M.S.); joel.patarinza@gmail.com (J.P.)
* Correspondence: jean.daou@uha.fr; Tel.: +33 3 89 33 67 39.

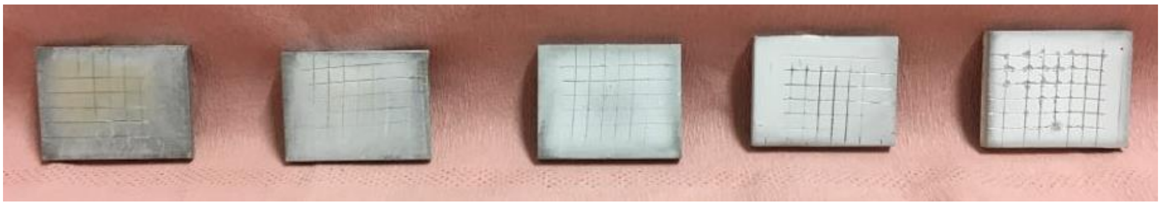

Figure S1. Adhesion test performed on zeolite coatings FAU-HK46 1 to 5 (from left to right).

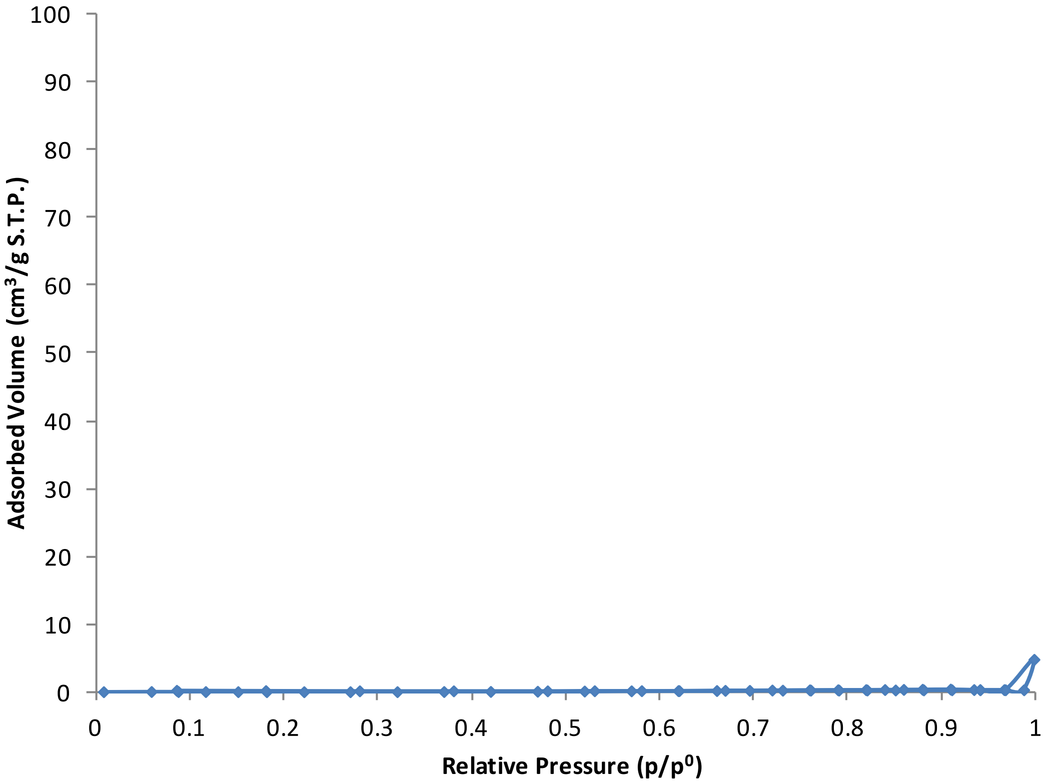

Figure S2. Nitrogen adsorption-desorption isotherms of the polymerized commercial binder SILRES® HK 46
